# Accessing the Microscopic World

**DOI:** 10.1371/journal.pbio.0030012

**Published:** 2005-01-18

**Authors:** Charles Carlson

## Abstract

The Exploratorium in San Francisco offers museum visitors the opportunity to use and manipulate state-of-the-art microscopes to visualize an array of living specimens

The Exploratorium, based in San Francisco, is a “hands on” science museum filled with interactive science and art exhibits, as well as a laboratory for the research and development of innovations in science education. In the summer of 2004, the Exploratorium launched the most ambitious microscope facility ever created for general public use. This initial phase of the project gives visitors the ability to image living specimens, as well as control the instruments themselves. Visitors can select among various specimens, move over them, change the magnification and focus, and, where appropriate, change the lighting to illuminate through the specimen, or use reflected light and fluorescence to dramatically change how it looks. They can image and explore tiny zebrafish embryos from the first stages of development to two-day-old fry with beating hearts and circulating blood cells, as well as a host of other organisms and cells from crawling amoebas to human blood cells.

Below the surface, all living things share common features. The primary goals of this facility are to open a door on the wonder of the microscopic world to a diverse range of museum visitors and allow them to explore it, and to allow them to make connections to science and biomedical research. By empowering visitors with the instruments to explore this unfamiliar universe, the Exploratorium seeks to recreate some of the excitement and wonder that the earliest researchers found as they discovered another world all around them (Box 1).

**Box 1. History of Light Microscope**

The light microscope falls amongst the greatest inventions of human history. Images from it in the 17th century literally revolutionized our understanding of life, providing first-hand evidence of a previously unseen or unsuspected world of organisms and cells all around us. This knowledge profoundly shaped our view of life, and of our placement in the universe. Robert Hooke used a primitive early microscope to see the walls between cells in a piece of cork (essentially discovering the cellular nature of all life), and Anton van Leeuwenhoek's simple scope revealed a previously unknown world of microorganisms living inside his own mouth. Swimming sperm were observed in semen, changing our fundamental understanding of conception. Since that time our world has become populated by marvelously beautiful and intriguing images and movies created by scientists using precise lighting and optics. Most recently, computer-controlled image-capturing techniques and digital technologies capture events and processes too small, slow, or fast for our unaided eyes to see.

Van Leeuwenhoek's first microscopes were probably about as powerful as a simple water-drop scope (see Box 2); he used his simple devices to make observations that weren't confirmed for over a hundred years. It's quite possible that early microscope-makers were inspired to make small domed lenses by observing the magnifying properties of a rounded drop of water. Further experimentation with the sizes and shapes of lenses eventually led to much greater magnifications.

## Beyond the Armored Microscope

Bringing a first-hand, high-quality microscope experience to a wide range of visitors and students has been a major challenge for many science museums and classrooms.

In the expanded, frenetic classroom of the science museum exhibition floor, microscopes themselves are too delicate and precise for operation without assistance and supervision: the optics can be easily damaged, all but the most robust of specimens are easily destroyed, and the user interface with lighting, positioning, and focus compose a world unfamiliar to most novice users. Over the years, we had collected our share of failed and broken microscopes in various projects. For these reasons most microscopes in museums have been armored, stripped-down, single-magnification instruments.

Problems related to the operation of the microscope hardware, however, represent only some of the challenges created by bringing uninitiated visitors to the microscopic world. The experience has to be repeatable. It has to be engaging. It has to be both simple and complex. It needs to work for individuals and groups. It needs to support investigation as well as provide essential information.

An array of research scientists joined us as we began work to redefine and renew our existing presentation on biology, most notably Christian Sardet, a cell biologist with a passion for visualization imagery. During the summer and fall of 1999, we engaged in a series of conversations directed at defining and developing a publicly accessible microscope imaging station, and the more we explored, the more technically feasible the project became.

At the same time, research-grade microscopes with high-quality optics underwent a revolution of their own. Many manufacturers planned to introduce fully automated, computer-controllable microscopes in their next models, and the prospects of having an off-the-shelf remotely controllable microscope seemed to solve a major hardware problem. It seemed as if software programs might be used to address many other user interface issues. In 2000, we applied for and received funding from the National Institutes of Health (a Science Education Partnership Award from the National Center for Research Resources) and from the David and Lucile Packard Foundation to support the development of a microscope imaging facility.

Seven months later we obtained the first microscope and its associated imaging equipment. But the software to control the microscopes proved dauntingly problematic. Programs that worked well in the laboratory proved difficult to adapt for our educational uses. Instead, we wrote our own programs to control stage movement (for specimen positioning), focus, specimen selection, magnification, and lighting. Each of these controls needed to be selectable and limited so that visitors might operate the microscope within a defined range.

On a parallel track, with the generous help of numerous biomedical researchers and their laboratories across the United States, we explored specimens for their visitor attraction and interest. Not surprisingly, we found that familiar structures and organisms provided the best entry points. A zebrafish embryo with a beating heart, circulating blood, and twitching tail movements rated more popular than zebrafish or sea urchin embryos at earlier stages of development. The tiny transparent roundworm Caenorhabditis elegans attracted attention with its earthworm-like movements. Overall, these biomedically relevant specimens provided a treasure trove of potential educational activities for visitors.

**Figure 1:**
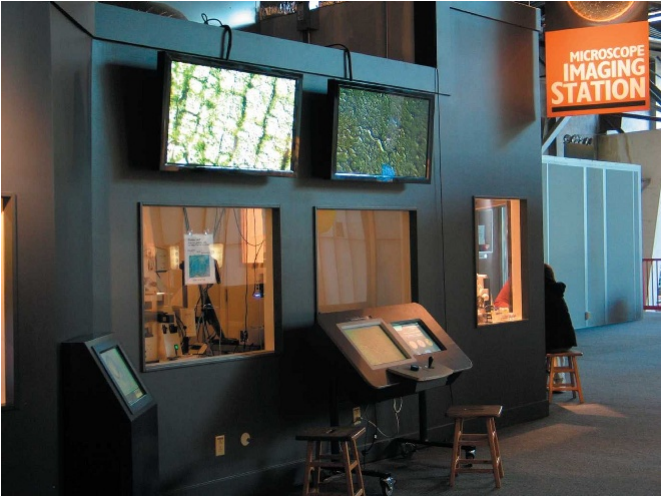
The Microscope Imaging Station Plasma screens, visitor microscope control consoles, and interactive media presentations provide visitors with access to live specimens and biomedical information

Given a range of wonderfully attractive specimens and potential activities, we then aimed to create an educational experience that combined the observable features in the specimens, supporting information, and further activities to be chosen as desired by the visitor. To achieve this, we adopted a multimedia approach (Figure 1). The incorporation of multimedia into the user–microscope interface required a complex piece of technology: the melding of live video imagery from the microscope with an interactive multimedia touchscreen, where selected microscope controls and specimen information changed as needed. To create this information presentation device, which we fondly refer to as the Usercart, we decided to use two side-by-side monitors with physical control devices for specimen position, focus, and variable magnification. Subsequent evaluation shows that the Usercarts work well; visitors get it!

Visitors can pick and choose specimens of their choice, refine their selection, change magnification, and engage in suggested activities at their own pace with integrated appropriate information. Individuals and groups engage with the microscope imagery and companion information for relatively long periods of time, ranging from several minutes to upwards of 20 minutes, a long time for a museum exhibit. To expand the use of the microscope imagery beyond that of one or two visitors, we incorporated large plasma-screen presentations in to the Microscope Imaging Station facility.

## What Else Is New?

In addition to using some of the latest devices in microscope technologies, the Imaging Station also provides a window on revolutionary research techniques. For example, in 1994, Columbia University's Martin Chalfie inserted a gene for a fluorescent jellyfish protein into a bacterium (Escherichia coli) and a roundworm (C. elegans) and found that the genetically modified organisms emitted an eerie green glow under certain conditions. Other scientists built on this technique to create a powerful tool that makes hidden structures and processes easier to study. The coupling of vital staining with the green fluorescent protein gene allows scientists to observe events inside developing cells and detect the presence of diseased structures and environmental toxins with extreme sensitivity. This newly created sensitivity has sparked new insights and discoveries, re-revolutionizing the capabilities of the light microscope.

At the Imaging Station, visitors can take advantage of this technique to observe specific types of cells—such as the brain cells, sex cells, or muscle cells of roundworms. Visitors can also peer inside the developing embryo of a tropical zebrafish whose circulatory cells have been made visible by the protein made from the transplanted green fluorescent protein gene. In the immediate future, visitors will be able to closely examine living human blood cells and fruit flies that researchers use to study the genetics of a wide range of human disorders. Over the next year, we intend to add a major component on mouse stem cells and the process of differentiation. At the Imaging Station, visitors have access to seamless video footage of events that take place in a fraction of a second or occur slowly over weeks or months (Box 1). Using the latest technologies available, we have built operating software specifically for the Imaging Station that gives visitors the same kind of control that professional researchers have over their own work. The straightforward user interface is integrated with explanatory graphics to provide control and orientation simultaneously. Finally, images are brought to users on large, high-resolution video screens. But the best may be yet to come, because the Imaging Station's capabilities are continually expanding, and visitors will ultimately be able to observe specimens by logging on to the Station's Web site (http://www.exploratorium.edu/imaging_station).

## Supporting Information

Video S1 A Medley of Time-Lapse Videos Collected at the Microscope Imaging Station Many of the cells and organisms found in these movies are available via visitor-accessible microscopes on a daily basis. At the station, visitor-directed, microscope-based observations form the basis for informal education on basic and biomedically relevant research. Video microscopy and production by Kristina Yu.
(5.4 KB MOV).

**Box 2. How to Make a Simple Microscope from a Drop of Water**

Materials. You'll need the clear plastic part of a CD case; if you can't find one, any thin sheet of rigid, clear plastic will work. Remove any paper or packaging so that you can see through the plastic. (Take the CD out, too!)

What to do. (1) Put the object you want to examine—a bug, a tiny leaf or plant, the small lettering on a coin or newspaper—on a flat surface. (2) Put one large drop of water on the center of the clear plastic. (An eyedropper makes this easy.) (3) Pick up the plastic and hold it horizontally so that the water drop is directly over the object. Move the plastic slowly back and forth to center the object, and up and down to focus. The water will form a curved shape that magnifies the object!

